# Adaptative Potential of the *Lactococcus Lactis* IL594 Strain Encoded in Its 7 Plasmids

**DOI:** 10.1371/journal.pone.0022238

**Published:** 2011-07-18

**Authors:** Roman K. Górecki, Anna Koryszewska-Bagińska, Marcin Gołębiewski, Joanna Żylińska, Marcin Grynberg, Jacek K. Bardowski

**Affiliations:** 1 Institute of Biochemistry and Biophysics, Polish Academy of Sciences, Warsaw, Poland; 2 Department of Biotechnology, Nicolaus Copernicus University, Toruń, Poland; University of Kansas Medical Center, United States of America

## Abstract

The extrachromosomal gene pool plays a significant role both in evolution and in the environmental adaptation of bacteria. The *L. lactis* subsp. *lactis* IL594 strain contains seven plasmids, named pIL1 to pIL7, and is the parental strain of the plasmid-free *L. lactis* IL1403, which is one of the best characterized lactococcal strains of LAB. Complete nucleotide sequences of pIL1 (6,382 bp), pIL2 (8,277 bp), pIL3 (19,244 bp), pIL4 (48,979), pIL5 (23,395), pIL6 (28,435 bp) and pIL7 (28,546) were established and deposited in the generally accessible database (GeneBank). Nine highly homologous *repB*-containing replicons, belonging to the lactococcal theta-type replicons, have been identified on the seven plasmids. Moreover, a putative region involved in conjugative plasmid mobilization was found on four plasmids, through identification of the presence of *mob* genes and/or *oriT* sequences. Detailed bioinformatic analysis of the plasmid nucleotide sequences provided new insight into the repertoire of plasmid-encoded functions in *L. lactis*, and indicated that plasmid genes from IL594 strain can be important for *L. lactis* adaptation to specific environmental conditions (e.g. genes coding for proteins involved in DNA repair or cold shock response) as well as for technological processes (e.g. genes encoding citrate and lactose utilization, oligopeptide transport, restriction-modification system). Moreover, global gene analysis indicated cooperation between plasmid- and chromosome-encoded metabolic pathways.

## Introduction

The lactic acid bacteria (LAB) are of great importance in food, agricultural, biotechnological and even pharmaceutical industries, which has made their physiology and genetics the subject of intensive studies. It is important to highlight that *L. lactis* is one of the most extensively used starter cultures in the LAB group, for production of various fermented dairy products such as cheese, sour cream and fermented milks [1]. Typically, *Lactococcus lactis* strains possess an abundance of plasmid DNA. Despite the fact that plasmids are generally dispensable for host survival, they often carry genes that might be essential for adaptation to extreme environmental conditions, such as genes encoding antibiotic and heavy metal resistance [2], [3], bacteriocins [2] or restriction-modification systems [4]. Moreover, some plasmids carry genes that are of technological interest, like genes encoding citrate utilization [5] and amino acid or sugar transporters [6]. These properties contribute to the desired flavor and texture of the product and to optimal growth in milk. It is for this reason that over the past 20 years a considerable effort has been put into characterizing *L. lactis* strains and their plasmids. Until recently, genomes of four *Lactococcus* strains [7]–[10] and thirty-five lactococcal plasmids have been completely sequenced (http://www.ncbi.nlm.nih.gov).

*L. lactis* subsp. *lactis* IL594 strain is the parental strain of the plasmid-free *L. lactis* IL1403, widely used in biological studies all over the world. In this paper, we present complete nucleotide sequences of the seven plasmids found in *Lactococcus lactis* IL594 cells, together with structural analysis of comparative and putative biological functions we assigned to them. Also is discussed plasmid replication, mobilization as well as the role of IS elements. Finally, we present a hypothesis claiming that the open reading frames carried by different plasmids or located on plasmids and on the chromosome form functional biological systems.

## Materials and Methods

### Bacterial strains, plasmids, and growth media

The bacterial strains and plasmids used in this study are listed in Table 1. *Lactococcus lactis* strains were obtained from the INRA collection, Jouy-en-Josas, France, and were grown at 30°C in M17 broth (Difco Laboratories, Detroit, MI) supplemented with 0.5% glucose or 0.5% lactose. *Escherichia coli* strain (TOPO10 Invitrogen) used as a host for the plasmid DNA fragments was grown in Luria-Bertani (LB) medium [14] at 37°C. When appropriate, ampicillin was added to a final concentration of 100 µg·ml^−1^ of *E. coli*. All bacteriological media were solidified with 1.5% agar when necessary.

**Table 1:** Lactococcal strains and phages used in this study.

*Lactococcus lactis* strains	Relevant characteristic(s)	Reference source
IL1403	plasmid-free host strain	[11]
IL594	harbouring seven plasmids named pIL1–pIL7	INRA France, [11]
IL1618	harbouring pIL1	
IL1392	harbouring pIL1, pIL2, pIL3, pIL5	
IL1421	harbouring pIL3	
IL2661	harbouring pIL4 (previously pIL9)	
IL1619	harbouring pIL5	
IL1420	harbouring pIL6	
IL1530	harbouring pIL7	
Lactococcal phages		
bIL67	c2-type representative phage	[12]
bIL170	936-type representative phage	[13]

### Phage assays

For phage plaque assay, 100 µl of serial dilutions of a phage stock (10^8–^10^9^ plaque forming units per ml, PFU/ml) were mixed with 200 µl of an overnight culture of the bacterial strain (Table 1) in 5 ml of the top GM17-0.75% agar and immediately poured onto GM17 plates containing 10 mM CaCl_2_. Plates were supplemented with erythromycin (5 µg/ml) when needed. The plates were checked for occurrence of phage plaques after incubation for 18 h at 30°C. Efficiency of plating (EOP) was calculated by dividing the number of PFU per ml for each phage plated on a phage-resistant tested strain by the number of PFU per ml on the sensitive indicator strain.

### DNA manipulations

Plasmid DNA was isolated by the alkaline lysis method followed by cesium chloride-ethidium bromide gradient ultracentrifugation [14]. DNA recombination and gel electrophoresis were performed according to standard procedures [14]. All enzymes used for DNA recombination and cloning were from MBI Fermentas, and enzymatic reactions were performed according to manufacturer's instructions.

### Construction of banks of DNA fragments

The shotgun libraries of the individual pIL3, pIL4, pIL5, pIL6 and pIL7 plasmid DNAs were prepared in the pCR4BluntTOPO plasmid with the use of the TOPO shotgun subcloning kit (Invitrogen).

The smallest plasmid, pIL1, extracted from the IL1618 strain (Table 1), was digested with *Eco*RI and the two resulting DNA fragments were ligated into *Eco*RI digested pBluescript SKII+ and cloned in the *E. coli* TOPO10 strain.

The DNA of plasmid pIL2 was recovered from the multiplasmid strain IL1392 by the following procedure. The plasmid DNA pool of IL1392 containing pIL1, pIL2, pIL3 and pIL5 was isolated and digested separately with several restriction enzymes. The obtained digestion patterns were compared with the digestion patterns of single plasmids pIL1, pIL3 and pIL5 treated with the same endonucleases. Only *Xba*I generated two unique fragments which were found to derive from pIL2. Both fragments of pIL2 were isolated by gel electrophoresis followed by electroelution from the agarose and subcloned into Bluescript SKII+ plasmid. Series of nested deletions of each fragment were generated using the ExoIII/Mung Bean deletion kit (Stratagene).

### Nucleotide sequence determination

pIL1 was sequenced by the primer walking method along the subcloned *Eco*RI fragments. In case of pIL2 the nested deletion clones of both fragments were sequenced using universal primers. Plasmids from pIL3 to pIL7 were sequenced using a shotgun sequencing approach according to the manufacturer's protocols; nucleotide sequences obtained were assembled and gaps were closed by primer walking, using oligonucleotides designed to contig ends. DNA sequencing and primers synthesis were performed by the DNA Sequencing & Oligonucleotide Synthesis Service at IBB PAS in Warsaw, using the Sequencer ABI377 (Applied Biosystems). When needed, single DNA sequencing reactions were performed at the bench using BigDye (Version 2.0; Applied Biosystems, Foster City, CA) and then analyzed in the service laboratory.

### Plasmid sequence assembly and annotation

Nucleotide sequences obtained were assembled with the use of the *Phred/Phrap/Consed* package and the *Consed* software autofinish function was used for gap closing and completion of the plasmid sequences [15]–[17]. For gene identification the Glimmer2 [18] as well as GeneMark for Prokaryotes (v 2.4) [19] programs were used; open reading frames (*orf*s) of >110 bp were retained. Homology searches of plasmidic *orf*s were performed by BLAST (Basic Local Alignment Search Tool) against the protein database through the NCBI website. Genes were functionally classified by assigning a “clusters of orthologous groups” (COG) number and corresponding COG category, DNA sequences were also analyzed using the Clone Manager 5 program (Scientific and Educational Software, Durham, N.C., USA).

For the prediction of transmembrane helices in proteins the TMHMM, (http://www.expasy.ch/tools) program was used.

The similarity of pIL plasmids to other plasmids was searched using the standard blastn program at the NCBI site [PMID: 2231712]. Best hits were used to show sequence similarity using Circoletto with a selected E-value of 0.1 [PMID: 20736339].

The complete nucleotide sequences of pIL1, pIL2, pIL3, pIL4, pIL5, pIL6 and pIL7 plasmids from *Lactococcus lactis* subsp. *lactis* IL594 were deposited in GeneBank under the accession numbers HM021326, HM021327, HM021328, HM021329, HM021330, HM021331 and HM197723, respectively.

## Results

It was suggested by Chopin *et al*. that *L. lactis* subsp. *lactis* IL594, the parental strain for IL1403, unequivocally contains nine plasmids [11]. However, in our present studies, we postulate that the IL594 strain possesses seven plasmids. In order to keep the sequence of digits from 1 to 7, the plasmid pIL9 carrying the lactose utilization genes was renamed pIL4. The complete nucleotide sequences of seven plasmids designated pIL1, pIL2, pIL3, pIL4, pIL5, pIL6 and pIL7 were determined, showing size range from 6 to 49 kb and GC content between 32.3 and 35.4% (Table 2); the latter is similar to other lactococcal plasmids and close to that of the chromosomal DNA of L. *lactis* subsp. *lactis* IL1403 (35.4%) [7]. However, several regions with different GC content were identified in five plasmids, pIL3–pIL7. The plasmid sequences have been annotated and analyzed in detail and are available as supplemental materials.

**Table 2:** Plasmids characteristics.

Plasmids	pIL1	pIL2	pIL3	pIL4	pIL5	pIL6	pIL7
Size in bp	6,382	8,277	19,244	48,978	23,395	28,435	28,546
GC content (%)	32.3	34.9	35.2	35.4	34.9	33.6	34.1
Total number of *orf*s	8	10	20	50	22	27	28
Homologs of proteins with known functions	3	5	10	37	17	12	17
Homologs of non lactococcal proteins	2	2	6	4	4	3	6

**Figure 1:**
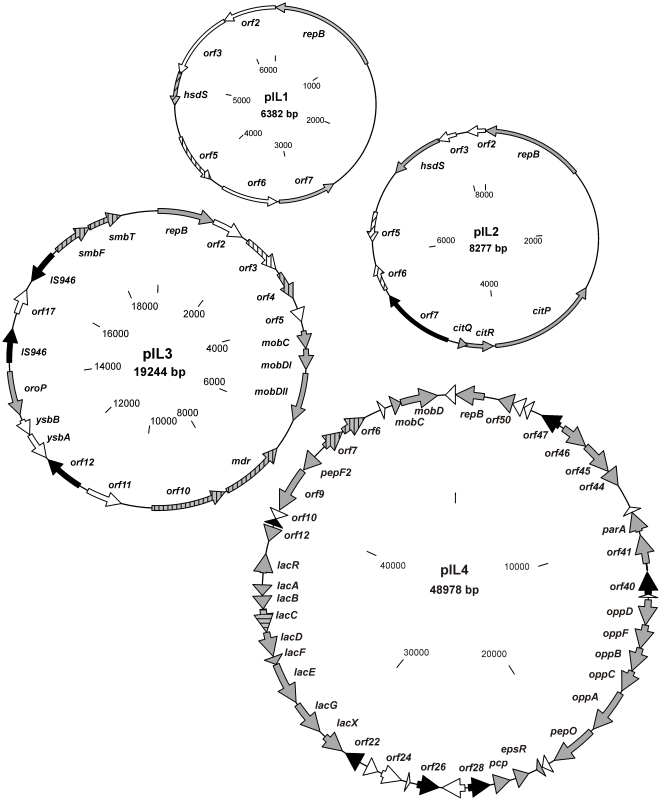
Physical and genetic maps of plasmids pIL1, pIL2, pIL3 and pIL4 of *L. lactis* IL594. All identified *orf*s are indicated by arrows showing the direction of transcription. The *orf*s with homologs of known fuction are colored grey, transposases and IS elements are in black and *orf*s homologous to other bacteria than lactococci are striped.

**Figure 2:**
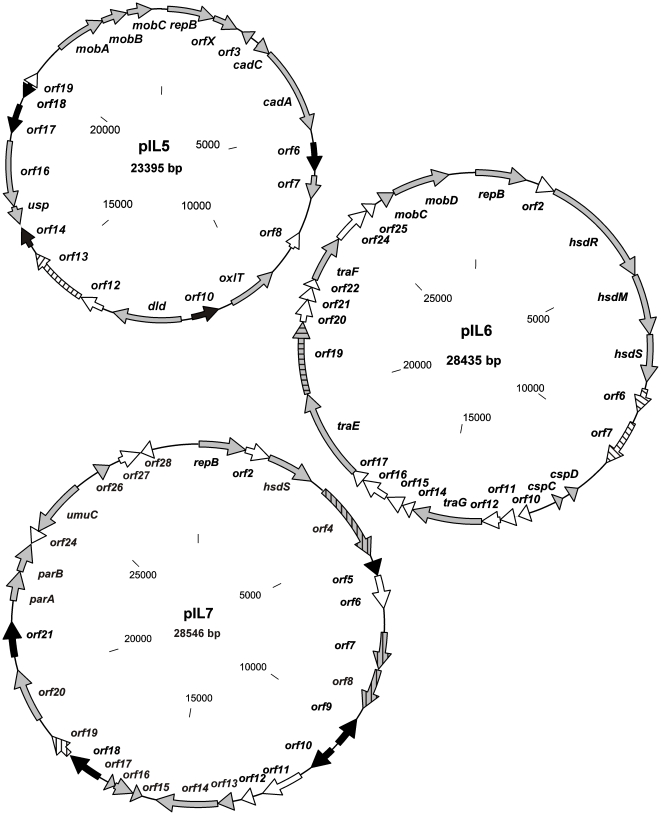
Physical and genetic maps of plasmids pIL5, pIL6 and pIL7 of *L. lactis* IL594. All identified *orf*s are indicated by arrows showing the direction of transcription. The *orf*s with homologs of known fuction are colored grey, transposases and IS elements are in black and *orf*s homologous to other bacteria than lactococci are striped.

Among the seven plasmids, spanning a total sequence of approximately 164 kb, 165 putative genes were identified (see Tables S1a–g in the supplemental material). Putative biological functions could be assigned to most of them. The majority of these genes displayed the highest degree of homology with genes located on the lactococcal chromosome or other lactococcal plasmids, but some shared close identity with genes from other, mainly Gram-positive bacteria, e.g. *Lactobacillus*, *Streptococcus*, *Listeria*, *Enterococcus* or *Carnobacterium*. Twenty IS elements, including two truncated elements that may mediate transposition events, were identified on all seven plasmids (Figures 1, 2).

### Sugar and organic acid utilization and regulation of internal pH

Intensive industrial use of lactococci has probably selected for strains which utilize the plasmid-encoded phospho-β-galactosidase. It was shown that cheese starters containing phospho-β-galactosidase rapidly fermented lactose to lactate, while non-dairy strains did it slowly. In the former case, lactose is taken up by the enzymes of the phophoenolopyruvate pathway (PEP-PTS), a system that catalyzes the formation of lactose-6-phosphate. On plasmid pIL4 were found genes encoding the PEP-PTS system and the tagatose-6-phosphate pathway responsible for the lactose-fermenting ability. These genes are organized in an operon with the gene order of *lacABCDFEGX* (*orf21-14*), while the transcriptional regulator LacR of the *lac* operon is positioned upstream (*orf13*) and in a divergent orientation to the operon. Rapid sugar conversion to lactate results in acidification of cytoplasm with an impact on membrane potential. Some metabolic features, due to plasmid genes, which might increase the pH gradient were also identified. The product of *orf10* localized on pIL2 shares 100% identity with the protein product of *citP* of the pCRL1127 plasmid (NP_258266). Upstream of *citP*, there are putative regulator genes *citQ* and *citR*. The *citP* gene encodes the citrate permease responsible for the uptake of divalent citrate with simultaneous transport of monovalent lactate out of the cell. For the *citQRP* operon, carried by another plasmid, pCIT264, the expression induced by acid stress due to lactate accumulation has been confirmed, underlying the significance of internal pH gradient for citrate permease expression [20]. *orf9* of pIL5 encodes a homolog of the oxalate/formate antiporter, OxlT, that includes 12 transmembrane helices and belongs to the major facilitator superfamily [21]. This protein carries out the electrogenic exchange of divalent oxalate with monovalent formate [22]. During such a transport (of both citrate and oxalate), generation of the proton-motive force occurs [23]. Both citrate and oxalate introduced to the cell by plasmidic transporters can be utilized by the bacterial cell. The metabolism of both substrates is carried out by chromosomally encoded enzymes.

*orf11* from plasmid pIL5, predicted to encode _D_-lactate dehydrogenase (D-LDH), also has an impact on the pH gradient. This putative FAD-dependent D-LDH is longer by about 200 amino acids from a typical NADH-dependent D-LDH generally found in other Gram-positive bacteria, including *L. lactis*. This NADH-independent D-LDH from *L. lactis* IL594 could function in D-lactate utilization under aerobic conditions and could have been acquired by this strain during milk fermentation processes. Consumption of D-lactate could contribute to pH reduction or lactate could be converted to acetate with production of ATP [24].

### Protein consumption

The ability to degrade casein and other milk proteins is also a very important feature of industrial strains since many dairy lactic acid bacteria are unable to synthesize certain amino acids necessary for their growth. The proteolytic system is composed of an extracellular proteinase, generally encoded by plasmid genes, specific transport systems and intracellular peptidases, engaged, respectively, in initial cleavage of casein resulting in small peptides and amino acids, in transport of oligopeptides into the cell, and, finally, in their hydrolysis to single amino acids [25]. *Orfs 38*-*33* from the pIL4 plasmid share ∼99% identity with the six genes encoding the whole oligopeptidase permease Opp system and endopeptidase PepO of *L. lactis* IL1403, SK11 and MG1363 strains [7]–[9].

As in the chromosome of *L. lactis* subsp. *lactis* IL1403, the six genes *oppDFBCApepO* form an operon in pIL4. The oligopeptide transport system is an ABC type transport composed of two transmembrane proteins, OppB and OppC, and two ATP-binding proteins, OppD and OppF. It has been confirmed that the Opp system plays an essential role in the nitrogen nutrition of *L. lactis*, especially in milk cultures. This gene cluster allows the lactococcal strain to transport and utilize oligopeptides generated by cell wall proteases, and leads to fast milk coagulation [26]. Moreover, upstream of the *opp* genes cluster, a predicted *pcp* gene encoding an N-terminal pyroglutamyl peptidase is present on pIL4. This component of the proteolytic system of *L. lactis* contributes to N-terminal degradation of peptides. The whole region, including *orf*s *39-29*, is flanked by two IS-like elements, suggesting that this segment had been transferred from the chromosome of *L. lactis* as a gene block. Furthermore, homologs of two other proteins involved in peptide utilization were found to be encoded by pIL4, since *orf9* and *orf8* encode, respectively putative oligopeptidases F and PepF2. Having multiple genes for certain proteolytic functions is an advantage for more efficient utilization of peptides derived from milk or present in other niches inhabited by *L. lactis*.

*orf16* carried by pIL5 codes for a protein that is not directly involved in peptide uptake, but might be crucial for the effectiveness of this process under certain conditions. This gene encodes a Mn^2+^ and Fe^2+^ transporter, which is similar to a chromosomally encoded protein of *L. lactis* SK11 and belongs to the NRAMP family (Natural Resistance-Associated Macrophage Protein). Such proteins pump divalent metal cations, mainly Mn^2+^ and Fe^2+^, and to lesser degree Co^2+^ and Cd^2+^, into the cytoplasm, using energy stored in ion electrochemical gradients [27]. Transport of divalent cations like Mn^2+^ and Fe^2+^ is critical since these metals are cofactors of both prokaryotic and eukaryotic catalases and superoxide dismutases [28]. Additionally, for bacteria consuming peptides the absence of Fe^2+^ near the cell surface is a necessity, since Fe^2+^ cations completely inhibit peptidase activity [29].

### Restriction modification system

RM systems are used by bacteria to protect themselves from a foreign DNA, such as that of bacteriophages. These systems are undoubtedly potent defense mechanisms against bacteriophages that cause contamination of starter cultures, resulting in bacterial cell lysis, spoilage and significant economic losses [30].

The gene products of *orf4*, *orf4*, *orf5* and *orf3* carried by pIL1, pIL2, pIL6 and pIL7, respectively were identified as S-subunits of type I restriction modification system. RM type I systems play a role in phage resistance due to digestion of incoming DNA unmethylated in a specific manner [31]. S-proteins are involved in DNA sequence recognition determining the specificity of methylase-endonuclease complexes [32]. The methylase and endonuclease subunits are encoded, respectively by chromosomal genes *hsdM* and *hsdR*. A typical S-protein has more than 400 residues and contains two conserved and two variable regions [33]. The conserved regions are responsible for interactions with M-subunits whereas the variable regions are engaged in sequence recognition [34]. In order to predict the domain structure of four HsdS proteins (HsdS_pIL1_, HsdS_pIL2_, HsdS_pIL6_ and HsdS_pIL7_), bioinformatic analyses of their amino acid sequences were carried out (data not shown). Results of these analyses showed that HsdS of pIL1 is truncated and consists of only 118 amino acids. HsdS_pIL2_ also seems to be truncated (217 amino acids) however its sequence analysis allowed to distinguish one conserved and one variable region. Thus, the HsdS_pIL2_ is unlikely to form an active complex with the methylase subunit, despite having one conserved region. However, the *hsdS* gene of pIL2 might be the source of variation in specificity due to homologous recombination with other *hsdS* genes, resulting in formation of chimeric S-subunits [35]. In case of HsdS_pIL6_ and HsdS_pIL7_ proteins, both of them consist of conserved and variable regions typical for functional S-subunits. Therefore, the phage resistance level of strain IL594 to lytic phages bIL67 and bIL170, belonging to different lactococcal bacteriophage genetic groups was determined. It was found that the IL594 strain was 10^4^-fold more resistant to both phages than the plasmid-free IL1403 strain (Table 3). Most known plasmids along with the three plasmids in this study (pIL1, pIL2 and pIL7) carry *hsdS* genes downstream of *orfs*, whose products exhibit homology to so-called replication-associated proteins (OrfX or RepX). pIL6 is a unique plasmid due to insertion of the *hsdR* and *hsdM* genes between *orfX* and *hsdS*. Both proteins are similar to lactococcal chromosomally encoded endonuclease and methylase.

**Table 3:** Efficiency of plating scores (EOPs)* for bIL67 and bIL170.

Bacteriophages		
Lactococcal strains	bIL67	bIL170
IL1403	1.00	1,00
IL594 (pIL1–pIL7)	1.06±0.1×10^−4^	3.9±0.25×10^−4^

*EOP (efficiency of plating): PFU (plaque forming units)×ml^−1^ formed on the indicator strain divided by PFU × ml^−1^ formed on the propagating strain. Data are means of at least three independent experiments.

### DNA damage repair

For a growing number of bacteria, the SOS response has been recognized as a critical component of the response to environmental stresses. The SOS response is the bacterial reaction to DNA-damaging chemical and physical factors and can be described as a rapid mobilization of the bacterial DNA repair system [36]. pIL7 was found to possess two *orf*s, *24* and *25*, predicted to encode proteins involved in DNA repair. The *orf25* encodes the UmuC-like protein, which is an essential component of the DNA damage mutagenesis mechanism in *E. coli* and possesses a DNA synthesis activity at the expense of normal replicative fidelity [36]. *orf24* is similar to a hypothetical protein predicted to be a member of the YolD protein family. Members of this family are functionally uncharacterized, but are postulated to represent functional equivalents to the UmuD subunit of DNA polymerase V (PolV). Both genes, *orf24* and *orf25*, are organized in the same way in pIL7 as in the chromosome of the IL1403 strain. *orf24* overlaps with *orf25* by six nucleotides, thus probably constituting an operon. The presence of an *umuC* ortholog in the lactococcal plasmid pNP40 was recently reported by O'Driscoll [37].

In addition, *orf15* from pIL5 encodes the universal stress protein UspA, a small cytoplasmic bacterial protein, whose expression is enhanced when the cell is exposed to stress agents. UspA protein enhances the rate of cell survival during prolonged exposure to stress conditions, and may provide a general “stress endurance” activity [38].

### Cold shock

*L. lactis* belongs to mesophilic microorganisms and during some industrial processes, like low temperature fermentation or food storage, strains of this species are exposed to low temperature, not optimal for their growth. Under such conditions they undergo important physiological changes. Cold shock proteins (CSPs) play an important role in overcoming the negative effects of temperature downshift during cold-shock adaptation [39]. The *L. lactis* subsp. *lactis* strain IL1403 [7] has two *csp* genes (*cspD* and *cspE*), whereas seven *csp* genes (*cspA* to *cspG*) have been found in *L. lactis* subsp. *cremoris* strain MG1363 [40].

Two *orf*s (*8* and *9*), found on pIL6, encode proteins that share identity with, respectively, LACR_C47 (100%) and LACR_C48 (98%) – the plasmid-encoded members of cold-shock DNA-binding protein family of *L. lactis* subsp. *cremoris* SK11 plasmid 3, as well as with chromosomally encoded cold shock proteins CspD (93%) and CspC (98%) of *L. lactis* subsp. *cremoris* MG1363. Interestingly, these two *orfs* are next to each other and in the same orientation in the *L. lactis* subsp. *cremoris* MG1363 chromosome as well as on both plasmids – pIL6 from IL594 and plasmid 3 from SK11 strains.

### Replication and maintenance system

**Figure 3:**
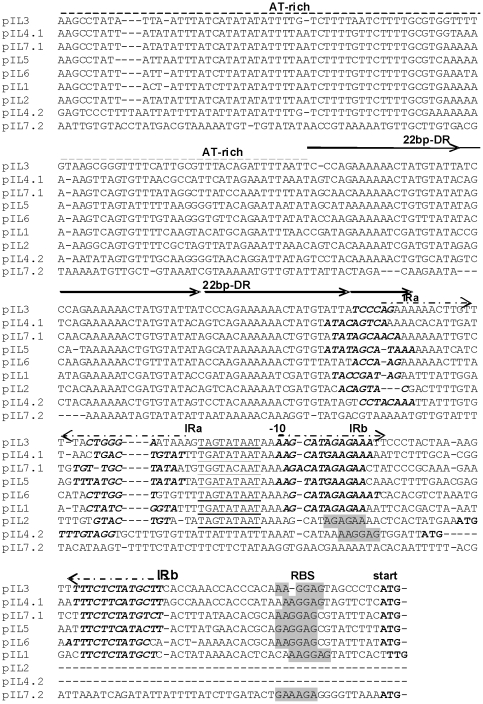
Multiple sequence alignment of upstream regions of *repB* genes. Indicated are the AT-rich regions, the 22-bp direct repeats, and in boldface the two inverted repeat regions (IRa, IRb). Extended promoter -10 site (consensus TGNTATAAT) is underlined, RBS is shaded. The ATG start codon of *repB* at the end of alignment is in bold.

In lactococci there are two types of plasmids, known as the rolling-circle replicating and theta-replicating, the latter appearing to be more frequent [41], [42]. Nine replicons have been identified on the seven plasmids, with two replicons found on each pIL4 and pIL7. All these replicons are characterized by the presence of a *repB* gene encoding the replication initiation protein RepB of 383 to 441 amino acid residues. The upstream noncoding regions of *repB* genes are highly conserved and correspond to those found in other lactococcal replicons, as first identified for plasmid pCI305 [41], [43]. Alignment analysis of the upstream regions of *repB* genes showed that plasmids present in the IL594 strain could replicate *via* a theta replication mechanism (Figure 3). All plasmids contain an AT-rich region that may serve as the recognition site for host-encoded functions involved in replication. This region is followed by a 22 bp sequence (DR; iterons) directly repeated three-and-half times, thought to interact directly with the RepB protein to initiate replication, and two inverted repeats, IRa and IRb. The IRa repeat, of 5 to 12 bp, overlaps in most regions with the last half iteron-repeat sequence. IRb repeat, of 11 to 13 bp, is found between the −10 box of the *repB* and the Ribosome Binding Site (RBS). The downstream region of the *repB* gene is often conserved in theta-type replicons as *repB-orfX-hsdS*. The *orfX* gene is usually overlapped by the *repB* gene by one or two codons, and thus in the majority of cases *orfX* seems to be translationally coupled to *repB*. While the presence of the *orfX* gene is very common among theta-replicating plasmids, it is not essential for replication. Indeed, several naturally occurring plasmids encode interrupted *orfX* genes and in others the gene is absent, as is the case for pWVO2 [42]. However, it has been shown that in some plasmids *orfX* does participate in the control of plasmid copy number, plasmid stability or both, but no such functions have been assigned with respect to lactococcal plasmids [41], [43], [44]. Such is also the case in *L. lactis* subsp. *lactis* IL594 strain, where *orfX* was found downstream of the *repB* in the seven of nine cases, while in one case, in the pIL1 plasmid, a hypothetical gene appeared to be inserted between *repB* and *orfX*; on pIL6 the same hypothetical gene is truncated. The *repB-orfX* genes are homologous to other lactococcal plasmid counterparts (77 to 98% amino acid identity). The last gene of this transcription unit encodes HsdS, the specifity subunit of a type I restriction modification system [45]. Intact or partial *hsdS* gene homologs, already described in the section “Restriction modification system”, are present on pIL1, pIL2, pIL6, and pIL7.

Plasmid replication is very closely linked to plasmid stability. Low-copy-number plasmids require stabilization mechanisms to prevent their loss during cell division. Such mechanisms include plasmid multimer resolution, postsegregational killing and an active partitioning system, all of which have been identified in both Gram-negative and Gram-positive bacteria [46]. pIL4 and pIL7 appear to contain determinants that contribute to their segregational stability. *orfs 22* and *23* from pIL7 are predicted to encode a complete partitioning cassette (ParA and ParB) most similar to ParA and ParB (∼99%) from the mobilizable lactococcal plasmid pGdh442 [47]. Moreover, *orf42* from pIL4 encodes an ATPase which is similar to the chromosome partitioning protein of *L. lactis* subsp *cremoris* SK11 [8]. However, those two ParA proteins present on pIL4 and pIL7 plasmids differ in size and when compared to each other share only 20% identity.

### Conjugation and mobilization

A large eighteen-gene-cluster mapped between cold shock (*csp*) and *repB* genes was identified on pIL6. Based on its sequence similarity to the appropriate region of the functional conjugative plasmid pNP40 [37], plasmid pIL6 may be able to transfer through the conjugation process. *orf27*, localized at the end of the cluster, codes for a protein homologous to MobD, which belongs to the Relaxase/Mobilization nuclease family that introduces a nick into duplex DNA [48]. However, no putative *oriT* has been found in the vicinity of the cluster on pIL6. The products of *orf13* and *orf18* reveal homology to TraG and TraE, respectively. TraG and TraE reveal similarity to components of type IV secretion system VirD4 and VirB4, respectively [48]. This fact may indicate the involvement of type IV secretion system in the conjugative transfer of pIL6. Type IV system is not only dedicated to conjugative transfer but also to filamentous bacteriophage secretion, protein secretion systems of several pathogens, and natural transformation [49].

*Orf19* encodes a TraG-related protein (pfam05257) corresponding to an amidase function. TraG is suggested to form a hole/break in the cell wall for the transmitted DNA, whereas the *orf24* product seems to protect such DNA against restriction in the recipient cell, because it possesses the antirestriction domain COG4227.

**Figure 4:**
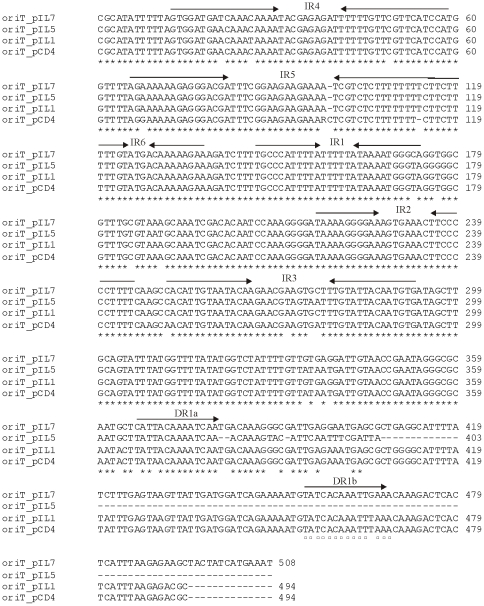
Sequence alignment of the *oriT* locus of lactococcal plasmids. Asterisks indicate identical nucleotides for all plasmids. Squares show identical nucleotides within the DR1B for three plasmids.

Moreover, sequence analyses of other plasmids of IL594 showed the presence of *mob-*like genes also on pIL3, pIL4, pIL5 and pIL7. Additionally, DNA sequences >95% identical to the transfer origin of pCD4 plasmid [50] were identified on plasmids pIL1, pIL5 and pIL7. Multiple alignment of the *oriT* sequences allowed to identify six inverted repeats and two direct repeats for pIL1 and pIL7, whereas for pIL5 only one direct repeat has been found (Figure 4). The *oriT* of plasmid pCD4 had previously been compared to the functional *oriT* of lactococcal plasmids pCI528 and pNZ4000 [51], [52]. In case of pIL1 and pIL7, such a region is localized upstream of the *repB* gene, whereas the *oriT* sequence of pIL5 is found upstream of *mob* genes, which code for proteins homologous to the nickase-relaxase family. The presence of both *mob* genes and *oriT* sequences might suggest a conjugative mobilization capacity of plasmids pIL1, pIL5 and pIL7.

## Discussion

During the past few years, considerable efforts have been made to better understand the molecular basis of genetic behavior of LAB, especially pertaining to their technological properties. We determined the complete nucleotide sequences of all seven plasmids present in the *L. lactis* IL594 strain.

Many industrially important traits like citrate, lactose and oligopeptides utilization and ions transport are plasmid encoded. As a consequence of this plasmid location the desired traits are lost when the plasmid is lost from the population. In this context, the presence of genes involved in a stable maintenance of the plasmid system (*parA*) on pIL4 and a complete partitioning cassette (*parAB*) found only on plasmid pIL7 might result in marked stability of pIL4 and pIL7 plasmids.

Another fact worth pointing out is the question of compatibility between the seven plasmids in the same cell. The principle, which says: the greater homology between two or more replicons is observed, the more likely they belong to the same incompatibility group and, therefore, are unlikely to be stably maintained in the same cell, is generally applied for plasmids of *Enterobacteriaceae*. In contrast to this, among lactococcal plasmids, such a strict correlation between sequence similarity of *ori* regions and plasmid incompatibility phenomenon is not observed [50]. Moreover, Gravesen raised the problem of the intellectual challenge of understanding the apparent paradox: how the lactococcal replicons being highly homologous can yet be compatible [52]. It has been suggested that the 22-bp tandem repeats DRII and/or the inverted repeat IRa in *ori* region together with a stretch of 13 amino acids of RepB are responsible for the plasmid-specific initiation of replication [50], [52]. Since each plasmidic fragment containing iterons and inverted repeats varies in nucleotide sequence (Figure 3) we assume that each *ori* region interacts specifically only with the corresponding RepB protein and thus, renders coexistence of seven plasmids in a bacterial cell of IL594 strain possible.

So far, the nucleotide sequences of LAB plasmids have been analyzed with respect to industrial applications of features encoded by them. Although many genes of technological importance have been identified on seven plasmids residing in *L. lactis* IL594, we focused on biological systems that result from global analysis of genes (Table 4). We proposed to distinguish three possible types of such interactions: (i) trail cooperation (a product of one metabolic pathway is a substrate for the second one), (ii) duplication (extra gene/region encoding protein revealing similar or identical function to the chromosomal one), and (iii) putative influence of plasmid regulatory proteins on chromosomal gene expression and *vice versa*. The proteins of *lac* operon, citrate and oxalate transporters, may be included in the type (i) interactions. The consumption of lactose, as well as of other sugars, leads to acidification of the interior of the cell resulting in *cit* operon expression. Citrate permease and oxalate transporters are engaged in an antiporter transport mechanism having impact on pH gradient. Moreover, acidification of the cell is also a signal for activation of chromosomally encoded citrate lyases. Metabolic transformation of citrate leads to an increase in membrane potential and yields a pH gradient due to proton consumption in the decarboxylation process [53].

**Table 4:** Some metabolic features of *Lactococcus lactis* IL594 encoded in twice - the chromosome and plasmid(s).

GENE LOCALIZATION								
FUNCTION	*L. lactis* chromosome	pIL1	pIL2	pIL3	pIL4	pIL5	pIL6	pIL7
Citrate metabolism	*citM-citCDEG*		*citQRP*					
Peptide utilization	*oppDFBCA, pepO, pepF*				*oppDFBCA, pepO, pepF, pcp*			
Restriction modification system I	*hsdRMS*		*hsdS*				*hsdRMS*	*hsdS*
DNA damage repair	*umuC*							*umuC*
Cold shock response	*cspD, cspE*						*cspC,cspD*	
Partition mechanism	*parA*			*parA*			*parAB*	

Cold shock proteins of pIL6 are an interesting variation of type (ii) predicted interactions. These proteins are very similar to chromosomally encoded members of *csp* family from *L. cremoris*. For this reason it is of interest, whether the extra *csp* genes introduced by pIL6 can *in trans* enhance the original effect (chromosomally encoded) of cryotolerance in *L. lactis* cells. The same kind of interaction-duplication of similar genes relates to chromosomally localized *umuC*, as a copy of this gene was found on pIL7 (*orf25*). It is also possible that the presence of the *opp* operon on pIL4, which is almost identical to the chromosomal one, gives rise to increased protein metabolism (e.g. casein).

The restriction modification system type I is an example of a potential plasmid∶chromosome genes interaction. We suppose that chromosomally encoded HdsM and HsdR subunits act together with HsdS encoded either by chromosomal or by plasmidic genes. Obtained result of phage resistance test suggests that HsdS proteins encoded by *orf5* of pIL6 and *orf3* of pIL7 can create functional holoenzymes with chromosomally encoded HsdR and HsdM resulting in the high level phage resistance of the IL594 strain. In case of pIL6 the situation is complex, because it encodes HsdM and HsdR proteins, which share high similarity with the chromosomal copies. It is speculated that an extra copy of *hsdRM* genes carried by pIL6 increases the level of phage resistance in case of the IL594 strain and hence, in *L. lactis*.

Most of the features described above are encoded by genes whose protein products share homology with proteins encoded by chromosomal genes of *L. lactis* subsp. *lactis* or *cremoris*. Most of *orf*s of unknown functions reveal almost 100% homology at amino acid level to chromosomal genes of *Lactococcus lactis*, suggesting some plasmid∶chromosome recombination events.

**Figure 5:**
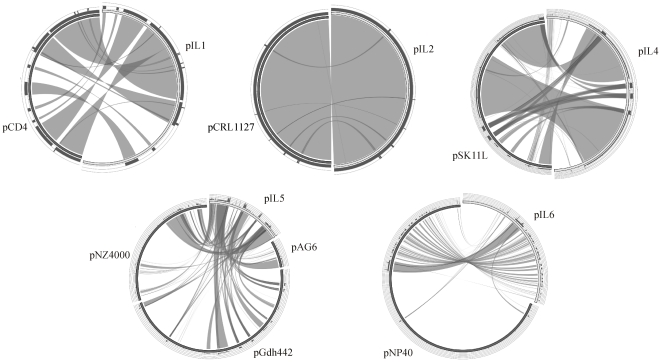
Sequence similarity between pIL plasmids and other lactococcal plasmids. Sequences of plasmids are placed around a circle. Names of plasmids are shown on the sides of each plasmid. Inside the circle, ribbons represent the local alignments BLAST has produced, in four shades of black, representing the four quartiles up to the maximum score - i.e. a local alignment with a score of 80% of the maximum score will be dark black, while one with 20% of the maximum score will be light grey. Ribbons may overlap and produce a rather complex picture. Included is a histogram on top of the ideograms, counting how many times each intensity of black has hit the specific part of the sequence. Also, a ribbon will invert if the local alignment is inverted, i.e. if the query hit the other strand of a database sequence, and will be outlined in black (compared to light grey) if it represents the best hit of the specific query against the database [PMID: 20736339].

Plasmids of *L. lactis* IL594 present a mosaic structure with sequence similarities to other lactococcal plasmids (Figure 5). Usually these regions cover operons or clusters involved in specific functions. The smallest two plasmids pIL1 and pIL2 are highly similar, respectively, to plasmids pCD4 and pCRL1127, both nucleotide sequences and genetic maps. Similarity between pIL1 and pCD4 covers more than 65% of the pIL1 length. The main difference is observed in the regions containing *hsdS* gene, since *hsdS* gene of pIL1 is truncated and an extra *orf* appeared in comparison to corresponding fragment of pCD4. The sequence and length of pIL2 were found to be almost identical (99.9%) with the pCRL1127 plasmid. Similarity between pIL4 and pSK11L plasmids is also quite significant and covers 65% of the pIL4 sequences including *repB* and two big operons like *opp* and lactose utilization. pIL5 is similar to pAG6, pGdh422 and pNZ4000 plasmids. pAG6 covers 31% of pIL5 sequence and encompasses the replication- and cadmium efflux-associated regions. pNZ4000 covers 36% and is similar in the *rep* and *mob* regions. pGdh422 covers 38% and is mainly similar to cadmium and lactose regions. The structure of plasmid pIL6 seems to be a combination of fragments derived from pAH82 and pNP40 plasmids. Similarity between pIL6 and pNP40 plasmids is quite significant and covers 53% of the pIL6 sequence. The region of high similarity to pNP40 ranges from cold shock operon (*orfs 8* and *9*) through genes of putative conjugation system to *mobDII*. Plasmids pIL3 and pIL7 are unique and seem to have no similar fragments with other lactococcal plasmids.

On the other hand, we identified a few *orf*s showing homology to genes residing in other bacterial genera. Among these are: (i) *mdr* and copper oxidase genes similar to chromosomal genes of *Lactobacillus*, (ii) clusters of genes localized independently on pIL7 and pIL4, almost identical to a chromosomal cluster from *Enterococcus faecalis*, (iii) the *orfs19*-*20* of pIL3, products of which exhibit homology to SmbF and SmbT proteins of *Streptococcus mutans*. The “enterococcal clusters” of pIL7 and pIL4 encode a membrane protein and a potential 2-dehydropantoate 2-reductase required for the synthesis of thiamine via an alternative pyrimidine biosynthetic pathway, whereas the *smbF* and *smbT* genes harbored by pIL3 are part of a nine-gene-operon coding for streptococcal bacteriocin synthesis and their products are involved in the transport of the prebacteriocin [54].

Transposition plays a significant role in plasmid∶chromosome interactions, whereas the conjugation phenomenon, mostly across species but also genera, contributes to the spread of plasmids. It is also speculated that a putative conjugation apparatus of pIL6 can be involved in its own transfer, as well as is able to induce conjugative transfer of other plasmids, on which putative *oriT* sequences have been found.

Noticeably, gene transfers occurred between bacterial genera living in the same biotype, such as *Lactobacillus* and *Streptococcus*, as previously reported for other *L. lactis* plasmids [3]. Those bacterial genera are present in both plant and dairy niches [55], probably because they can transfer from forage plants and meadow grasses to milk via cattle, and vice versa. For *L. lactis* subsp. *lactis*, plant-derived strains are thought to be the natural source of milk derived strains [55].

Plasmids are common components of the genome of *L. lactis* strains and encode valuable properties ensuring their predominance in the dairy environment. In this work the nucleotide sequences of seven plasmids present in the *Lactococcus lactis* IL594 strain were established and annotated in the GeneBank. The presence of complete IS elements, genes sharing very close identity with genes from other bacteria, as well as several regions with different GC content suggest a mosaic structure of the plasmids. Analysis of the genetic structure of seven plasmids from the IL594 strain in relation to the chromosome of IL1403 led us to propose a putative cooperation between them, suggesting the existence of a systemic cooperation in biology.

## Supporting Information

Table S1 a: Putative genes identified on pIL1, b: Putative genes identified on pIL2, c: Putative genes identified on pIL3, d: Putative genes identified on pIL4, e: Putative genes identified on pIL5, f: Putative genes identified on pIL6, g: Putative genes identified on pIL7.
(DOC)
