# Supplementary material for: Adaptative Potential of the Lactococcus Lactis IL594 Strain Encoded in Its 7 Plasmids
Source: PLoS One. 2011 Jul 18;6(7):e22238. doi: 10.1371/journal.pone.0022238 (PMC3138775; doi:10.1371/journal.pone.0022238)
Supplement: Table S1 — a: Putative genes identified on pIL1, b: Putative genes identified on pIL2, c: Putative genes identified on pIL3, d: Putative genes identified on pIL4, e: Putative genes identified on pIL5, f: Putative genes identified on pIL6, g: Putative genes identified on pIL7. (DOC) [file pone.0022238.s001.doc]

**SUPPLEMENTAL MATERIAL**

**Table S1a: Putative genes identified on pIL1.**

| Tag | Gene | Position | Length  (AA) | Best blast match | Transmembrane domains | Gene Bank accession no. | AA overlap (identity %) | Organism, plasmid |
| --- | --- | --- | --- | --- | --- | --- | --- | --- |
| pIL1_1 | *repB* | 1155...1 | 285 | replication initiator protein RepB |  | NP_112721 | 386 (88) | *L. lactis*, pCD4 |
| pIL1_2 | *orf2* | 6382...5839 | 169 | hypothetical protein LACR_B7 | 5 | YP_796467 | 175 (97) | *L. lactis* subsp. *cremoris* SK11 |
| pIL1_3 | *orf3* | 5843...5112 | 244 | hypothetical protein pDR11_02 |  | NP_690630 | 214 (86) | *L. lactis*, pDR11 |
| pIL1_4 | *hsdS* | 5133...4777 | 104 | type I restriction-modification system specificity subunit, truncated |  | YP_139340 | 108 (92) | *S. thermophilus* |
| pIL1_5 | *orf5* | 4439...3894 | 80 | XRE family transcriptional regulator | 3 | YP_819422 | 180 (48) | *L. mesenteroides* |
| pIL1_6 | *orf6* | 3749...3170 | 193 | unknown, contains short-chain alcohol dehydrogenases motif | 5 | AAC15899 | 193 (93) | *L. lactis*, pCD4 |
| pIL1_7 | *orf7* | 3151...2564 | 196 | tyrosine family DNA recombinase |  | NP_112720 | 196 (98) | *L. lactis*, pCD4 |

**Table S1b: Putative genes identified on pIL2.**

| Tag | Gene | Position | Length  (AA) | Best blast match | Transmembrane domains | Gene Bank accession no. | AA overlap (identity %) | Organism, plasmid |
| --- | --- | --- | --- | --- | --- | --- | --- | --- |
| pIL2_1 | *repB* | 1206...1 | 385 | replication protein RepB |  | NP_258267 | 385 (100) | *L. lactis* |
| pIL2_2 | *orf2* | 8277...8061 | 74 | hypothetical protein |  | NP_258268 | 74 (100) | *L. lactis*, pCRL1127 |
| pIL2_3 | *orf3* | 7932...7708 | 75 | hypothetical protein |  | NP_053008 | 209 (96) | *L. lactis* subsp. *cremoris*, pNZ4000 |
| pIL2_4 | *hsdS* | 7717...7068 | 217 | putative type IS subunit protein |  | NP_258262 | 217 (100) | *L. lactis* |
| pIL2_5 | *orf5* | 6535...6167 | 121 | hypothetical protein |  | YP_002474158 | 226 (56) | *Lactobacillus gasseri* |
| pIL2_6 | *orf6* | 5557...5883 | 69 | hypothetical protein |  | YP_002268570 | 75 (50) | *Lactobacillus casei* |
| pIL2_7 | *orf7* | 4586...5473 | 296 | putative IS-like element |  | NP_258263 | 296 (100) | *L. lactis*, pCRL1127 |
| pIL2_8 | *citQ* | 4450...4349 | 33 | putative translational regulator CitQ |  | NP_258264 | 33 (100) | *L. lactis,* pCRL1127 |
| pIL2_9 | *citR* | 4382...4044 | 112 | putative translational regulator CitR |  | NP_258265 | 112 (100) | *L. lactis,* pCRL1127 |
| pIL2_10 | *citP* | 4018...2693 | 442 | citrate permease/transporter CitP | 13 | NP_258266 | 442 (100) | *L. lactis*, pCRL1127 |

**Table S1c: Putative genes identified on pIL3.**

| Tag | Gene | Position | Length  (AA) | Best blast match | Transmembrane domains | Gene Bank accession no. | AA overlap (identity %) | Organism, plasmid |
| --- | --- | --- | --- | --- | --- | --- | --- | --- |
| pIL3_1 | *repB* | 4...1185 | 393 | replication protein |  | NP_690629 | 423 (82) | *L. lactis* |
| pIL3_2 | *orf2* | 1181...1849 | 223 | hypothetical protein pSRQ700_02 |  | NP_116727 | 209 (36) | *L. lactis*, pSRQ700 |
| pIL3_3 | *orf3* | 1991...2815 | 269 | hypothetical protein CAT7_02899 | 6 | ZP_02184849 | 274 (47) | *Carnobacterium* sp. AT7 |
| pIL3_4 | *orf4* | 2924...3511 | 195 | putative resolvase |  | CAB46556 | 198 (88) | *Streptococcus thermophilus* |
| pIL3_5 | *orf5* | 3748...4026 | 78 | unkonown |  | NP_862607 | 105 (54) | *L. lactis*, pAH82 |
| pIL3_6 | *mobC* | 4221...4586 | 122 | mobilisation protein (MobC) |  | YP_796489 | 122 (92) | *L. lactis* subsp.*cremoris* SK11 |
| pIL3_7 | *orf7* | 4594...5019 | 122 | relaxase/mobilization nuclease domain-containing protein (MobDI) |  | YP_796488 | 135 (90) | *L. lactis* subsp. *cremoris* SK11 |
| pIL3_8 | *orf8* | 5099...6103 | 335 | relaxase Mob DEII |  | NP_862611 | 256 (98) | *L. lactis* subsp. *lactis* |
| pIL3_9 | *orf9* | 8140...6770 | 457 | major facilitator superfamily permease | 13 | YP_807596 | 457 (99) | *Lb. casei* |
| pIL3_10 | *orf10* | 9742...8183 | 520 | putative multicopper oxidase |  | YP_807597 | 509 (99) | *Lb. casei* |
| pIL3_11 | *orf11* | 11125...10379 | 249 | putative nicotinamide mononucleotide transporter | 8 | YP_001174683 | 249 (98) | *L. lactis* |
| pIL3_12 | *orf12* | 11322...12167 | 282 | putative IS-like element |  | NP_258263 | 296 (98) | *L. lactis*, pCRL1127 |
| pIL3_13 | *ysbA* | 12798...12220 | 193 | hypothetical protein YsbA |  | YP_001098364 | 193 (99) | *L. lactis* subsp. *lactis* bv. *diacetylactis* |
| pIL3_14 | *ysbB* | 13147...12806 | 114 | hypothetical protein YsbB |  | YP_001098363 | 114 (100) | *L. lactis* subsp. *lactis* bv. *diacetylactis* |
| pIL3_15 | *oroP* | 14182...13262 | 307 | orotate transporter | 9 | YP_001098362 | 307 (99) | *L. lactis*, pDBORO |
| pIL3_16 | *orf16* | 14371...15078 | 236 | insertion sequence IS946 transposase |  | ABW87264 | 236 (97) | *L. lactis* |
| pIL3_17 | *orf17* | 15401...15934 | 178 | hypothetical YreD | 4 | NP_267845 | 178 (98) | *L. lactis* |
| pIL3_18 | *orf18* | 16833...16126 | 236 | insertion sequence IS946 transposase |  | ABW87264 | 236 (96) | *L. lactis* |
| pIL3_19 | *smbF* | 16950...17774 | 257 | transportation and immunity of preSmb |  | BAD72772 | 274 (60) | *Streptococcus mutans* |
| pIL3_20 | *smbT* | 17767...18477 | 229 | transportation of preSmb | 6 | BAD72773 | 238 (52) | *Streptococcus mutans* |

**Table S1d: Putative genes identified on pIL4.**

| Tag | Gene | Position | Length  (AA) | Best blast match | Transmembrane domains | Gene Bank accession no. | AA overlap (identity) | Organism, plasmid |
| --- | --- | --- | --- | --- | --- | --- | --- | --- |
| pIL4_50 | *orf50* | 1146…547 | 199 | putative resolvase |  | AAF27558 | 199 (100) | L. lactis, pCI2001 |
| pIL4_49 | *orf49* | 1605…1150 | 151 | unknown protein | 1 | AAF27556 | 151 (100) | *L. lactis,* pCI2001 |
| pIL4_48 | *orf48* | 1932...1636 | 98 | hypothetical protein |  | AAF27316 | 98 (98) | *L. lactis,* pCI2001 |
| pIL4_47 | *orf47* | 2994...2404 | 196 | transposase |  | YP_808692 | 284 (99) | *L. lactis* subsp. *cremoris* SK11 |
| pIL4_46 | *nah* | 3433...4575 | 380 | Na+/H+ antiporter pGDH422 | 11 | YP_001174692 | 380 (98) | *L. lactis,* pGdh44 |
| pIL4_45 | *orf45* | 4592…5929 | 445 | putative K+ transporter pGDH422 | 11 | YP_001174693 | 445 (99) | *L. lactis,* pGdh44 |
| pIL4_44 | *orf44* | 5949…6617 | 222 | putative K+ transport system pGDH422 |  | YP_001174694 | 222 (100) | *L. lactis,* pGdh44 |
| pIL4_43 | *orf43* | 7818…7594 | 74 | hypothetical protein |  | ABA47347 | 74 (98) | *L. lactis* subsp. *cremoris* SK11, pSK11L |
| pIL4_42 | *orf42* | 8629…7820 | 269 | ATPase for chromosome partitioning |  | YP_796551 | 296 (99) | *L. lactis* subsp. *cremoris* SK11 |
| pIL4_41 | *repB1* | 9929…8775 | 384 | replication initiator protein |  | YP_796550 | 384 (99) | *L. lactis* subsp. *cremoris* SK11, pSK11L |
| pIL4_40 | *orf40* | 11097…10207 | 296 | transposase |  | YP_811441 | 296 (100) | *L. lactis* subsp. *cremoris* SK11 |
| pIL4_39 | *orf39* | 11282…11154 | 42 | hypothetical protein llmg_0696 |  | YP_001032035 | 57 (75) | *L. lactis* subsp. *cremoris* MG1363 |
| pIL4_38 | *oppD* | 11375…12391 | 338 | oligopeptide ABC transporter ATP binding protein |  | YP_796548 | 338 (100) | *L. lactis* subsp. *cremoris* SK11, pSK11L |
| pIL4_37 | *oppF* | 12388…13347 | 319 | oligopeptide ABC transporter ATP binding protein |  | NP_267997 | 319 (99) | *L. lactis* subsp*. lactis* IL1403 |
| pIL4_36 | *oppB* | 13328...14287 | 319 | oligopeptide ABC transporter permease protein | 6 | NP_267996 | 319 (100) | *L. lactis* subsp*. lactis* IL1403 |
| pIL4_35 | *oppC* | 14297...15181 | 294 | oligopeptide ABC transporter permease protein | 6 | NP_267995 | 294 (100) | *L. lactis* subsp*. lactis* IL1403 |
| pIL4_34 | *oppA* | 15291...17093 | 600 | oligopeptide-binding protein oppA precursor |  | YP_001032040 | 600 (100) | *L. lactis* subsp*. cremoris* MG1363 |
| pIL4_33 | *orf33* | 17218…19101 | 627 | oligopeptidase O1 |  | YP_796543 | 627 (99) | *L. lactis* subsp. *cremoris* SK11, pSK11L |
| pIL4_32 | *orf32* | 19348…19644 | 98 | conserved hypothetical protein |  | ABA47377 | 112 (100) | *L. lactis* subsp. *cremoris* SK11, pSK11L |
| pIL4_31 | *orf31* | 19679…19900 | 73 | EpsR |  | AAP32713 | 105 (40) | *L. lactis* subsp. *cremoris,* pCI658 |
| pIL4_30 | *orf30* | 20804…20250 | 184 | site-specific recombinase, DNA invertase Pin related protein |  | YP_796542 | 184 (100) | *L. lactis* subsp. *cremoris* SK11, pSK11L |
| pIL4_29 | *pcp* | 21696…21049 | 215 | pyrrolidone-carboxylate peptidase pSK11 |  | ABA47397 | 215 (99) | *L. lactis* subsp. *cremoris* SK11, pSK11P |
| pIL4_28 | *orf28* | 22761…21871 | 296 | transposase |  | YP_808889 | 296 (98) | *L. lactis* subsp. *cremoris* SK11 |
| pIL4_27 | *orf27* | 22896...23828 | 310 | hypothetical protein L124727 | 10 | NP_267058 | 310 (98) | *L. lactis* subsp. *lactis* IL1403 |
| pIL4_26 | *orf26* | 24828...23936 | 288 | transposase |  | YP_796512 | 296 (95) | *L. lactis* subsp. *cremoris* SK11, pSK11P |
| pIL4_25 | *orf25* | 25276…25136 | 46 | hypothetical protein |  | ZP_03980228 | 49 (95) | *Enterococcus faecium* |
| pIL4_24 | *orf24* | 26332…25487 | 281 | hypothetical protein |  | YP_001966461 | 281 (99) | *L. lactis,* pNP40 |
| pIL4_23 | *orf23* | 26982…26512 | 156 | hypothetical protein |  | YP_001966460 | 156 (100) | *L. lactis,* pNP40 |
| pIL4_22 | *orf22* | 27328…28008 | 226 | insertion sequence IS946 |  | NP_047328 | 226 (97) | *L. lactis,* pMRC01 |
| pIL4_21 | *lacX* | 29059…28154 | 301 | galactose mutarotase-like protein |  | YP_796563 | 301 (99) | *L. lactis* subsp. *cremoris* SK11, pSK11L |
| pIL4_20 | *lacG* | 30778…29345 | 477 | 6-phospho-beta-galactosidase |  | AAA26949 | 477 (99) | *L. lactis* |
| pIL4_19 | *lacE* | 32564…30856 | 548 | cellobiose-specific PTS system IIC component | 9 | YP_796530 | 568 (93) | *L. lactis* subsp. *cremoris* SK11, pSK11L |
| pIL4_18 | *lacF* | 32885…32568 | 105 | Lactose-specific phosphotransferase enzyme IIA component |  | AAA25181 | 105 (99) | *L. lactis* |
| pIL4_17 | *lacD* | 32913…33893 | 326 | tagatose 1,6-diphosphate aldolase |  | YP_796532 | 326 (100) | *L. lactis* subsp. *cremoris* SK11, pSK11L |
| pIL4_16 | *lacC* | 34828…33896 | 310 | tagatose-6-phosphate kinase [ |  | ZP_03980299 | 310 (100) | *Enterococcus faecium* |
| pIL4_15 | *lacB* | 35354…34839 | 171 | galactose-6-phosphate isomerase subunit LacB |  | YP_796534 | 171 (98) | *L. lactis* subsp. *cremoris* SK11, pSK11L |
| pIL4_14 | *lacA* | 35796…35371 | 141 | galactose-6-phosphate isomerase lacA subunit |  | AAA25168 | 141 (100) | *L. lactis* |
| pIL4_13 | *lacR* | 36275...37044 | 255 | lactose transport regulator |  | YP_796536 | 255 (89) | *L. lactis* subsp. *cremoris* SK11, pSK11L |
| pIL4_12 | *orf12* | 38124...37543 | 193 | site-specific recombinase, DNA invertase Pin related protein |  | YP_796537 | 193 (98) | *L. lactis* subsp. *cremoris* SK11, pSK11L |
| pIL4_11 | *yajE* | 38217...38477 | 86 | transposase |  | NP_266244 | 86 (100) | *L. lactis* subsp. *lactis* IL1403 |
| pIL4_10 | *orf10* | 38855...38694 | 53 | hypothetical protein |  | CAA68134 | 53 (100) | *L. lactis* |
| pIL4_9 | *pepF* | 40663…38858 | 601 | oligopeptidase F |  | NP_267883 | 601 (99) | *L. lactis* |
| pIL4_8 | *pepF2* | 41403…40726 | 225 | pepF2 |  | CAA68132 | 215 (96) | *L. lactis* |
| pIL4_7 | *orf7* | 41975…42193 | 312 | 2-dehydropantoate 2-reductase |  | NP_814296 | 312 (99) | *Enterococcus faecalis* |
| pIL4_6 | *orf6* | 42939…43976 | 345 | membrane protein, toxin regulator | 9 | NP_814295 | 345 (99) | *Enterococcus faecalis* |
| pIL4_5 | *orf5* | 44673…44870 | 65 | hypothetical protein pGdh442_p08 |  | YP_001174703 | 64 (93) | *L. lactis,* pGdh442 |
| pIL4_4 | *mobC* | 45176…45547 | 123 | MobC |  | YP_001174699 | 122 (90) | *L. lactis,* pGdh442 |
| pIL4_3 | *mobD* | 45550…47607 | 505 | MobD |  | YP_001966491 | 505 (99) | *L. lactis,* pNP40 |
| pIL4_2 | *orf2* | 47785…47375 | 136 | hypothetical protein pCI2001 |  | AAF27560 | 136 (100) | *L. lactis,* pCI2001 |
| pIL4_1 | *repB1* | 48944…47778 | 388 | putative replication protein pCI2001 |  | AAF27559 | 388 (100) | *L. lactis,* pCI2001 |

**Table S1e: Putative genes identified on pIL5.**

| Tag | Gene | Position | Length  (AA) | Best blast match | Transmembrane domains | Gene Bank accession no. | AA overlap (identity) | Organism, plasmid |
| --- | --- | --- | --- | --- | --- | --- | --- | --- |
| pIL5_1 | *repB* | 150…1301 | 383 | RepB |  | NP_053040 | 383 (99) | *L. lactis* subsp. *cremoris,* pNZ4000 |
| pIL5_2 | *orfX* | 1301…1921 | 206 | replication-associated protein RepX |  | YP_003329497 | 200 (52) | *L.* *lactis* subsp*. cremoris* SK11, pSK11B |
| pIL5_3 | *orf3* | 2314…2057 | 85 | peptidase E |  | YP_809513 | 231 (37) | *L.* *lactis* subsp*. cremoris* SK11 |
| pIL5_4 | *cadC* | 2543…2902 | 119 | cadmium resistance regulator CadC |  | NP_862601 | 119(100) | *L. lactis* subsp. *lactis,* pAH82 |
| pIL5_5 | *cadA* | 2899…5016 | 705 | cadmium efflux ATPase CadA | 5 | NP_862600 | 705 (99) | *L. lactis* subsp. *lactis,* pAH82 |
| pIL5_6 | *orf6* | 5327…6007 | 226 | transposase |  | AAC44536 | 226 (98) | *Leuconostoc mesenteroides* |
| pIL5_7 | *orf7* | 6114…6677 | 187 | glycopeptide antibiotics resistance protein | 6 | YP_796479 | 191 (99) | *L. lactis* subsp*. cremoris* SK11 |
| pIL5_8 | *orf8* | 7843…7526 | 136 | hypothetical protein llmg_0668 |  | YP_001032007 | 105 (97) | *L. lactis* subsp*. cremoris* MG1363 |
| pIL5_9 | *oxlT* | 9915…8674 | 413 | oxalate/formate antiporter | 23 | YP_001032006 | 418 (99) | *L. lactis* subsp*. cremoris* MG1363 |
| pIL5_10 | *orf10* | 10958…10278 | 226 | transposase |  | CAA64387 | 226 (97) | *Streptococcus thermophilus* |
| pIL5_11 | *dld* | 11227…12906 | 559 | D-lactate dehydrogenase |  | YP_796500 | 559 (99) | *L. lactis* subsp*. cremoris* SK11, pSK11P |
| pIL5_12 | *orf12* | 13163…13756 | 197 | unnamed protein product |  | CAA73267 | 200 (34) | *L. lactis* subsp*. cremoris,* pJW565 |
| pIL5_13 | *orf13* | 13778…15292 | 504 | hypothetical membrane protein | 7 | BAF47170 | 509 (32) | *Streptococcus mutans* |
| pIL5_14 | *orf14* | 15498…15989 | 163 | transposase |  | ZP_05231446 | 193 (86) | *Listeria monocytogenes* |
| pIL5_15 | *usp* | 16525…16088 | 145 | universal stress protein UspA |  | YP_796557 | 145 (98) | *L. lactis* subsp. *cremoris* SK11, pSK11L |
| pIL5_16 | *orf16* | 18111…16534 | 525 | manganese transporter NRAMP | 12 | YP_796556 | 525 (100) | *L. lactis* subsp. *cremoris* SK11, pSK11L |
| pIL5_17 | *orf17* | 19006…18302 | 234 | transposase |  | ABI_93963 | 273 (98) | *L. lactis,* pTR2030 |
| pIL5_18 | *orf18* | 19575…19168 | 135 | transposase |  | YP_796471 | 91 (100) | *L. lactis* subsp. *cremoris* SK11, pSK11P |
| pIL5_19 | *orf19* | 19560…19880 | 106 | hypothetical protein pSRQ800_07 |  | NP_862566 | 196 (98) | *L. lacti*s, pSRQ800 |
| pIL5_20 | *mobA* | 20689…21921 | 410 | mobilization protein |  | AAA99865 | 410 (99) | *L. lactis,* pCI528 |
| pIL5_21 | *mobB* | 21918…22541 | 207 | putative mobilization protein | 2 | NP_053038 | 207 (94) | *L. lactis* subsp*. cremoris,* pNZ4000 |
| pIL5_22 | *mobC* | 22558…23160 | 200 | putative mobilization protein |  | NP_053039 | 200 (99) | *L. lactis* subsp*. cremoris,* pNZ4000 |

**Table S1f: Putative genes identified on pIL6.**

| Tag | Gene | Position | Length  (AA) | Best blast match | Transmembrane domains | Gene Bank accession no. | AA overlap (identity %) | Organism, plasmid |
| --- | --- | --- | --- | --- | --- | --- | --- | --- |
| pIL6_1 | *repB* | 1...1335 | 445 | replication protein RepB |  | CAA65652 | 441 (86) | *L. lactis* |
| pIL6_2 | *orf2* | 1673...2106 | 99 | hypothetical protein LACR_E7 |  | YP_796571 | 224 (96) | *L. lactis* subsp. *cremoris* SK11 |
| pIL6_3 | *hsdR* | 2120...5195 | 1025 | restriction subunit |  | AAB91415 | 1025 (95) | *L. lactis* subsp. *lactis* bv. diacetylactis |
| pIL6_4 | *hsdM* | 5198...6790 | 531 | putative type I site-specific deoxyribonuclease |  | YP_001032001 | 545 (99) | *L. lactis* subsp. cremoris MG1363 |
| pIL6_5 | *hsdS* | 6786...8027 | 414 | restriction modification system subunit HsdS |  | NP_862616 | 421 (42) | *L. lactis*, pAH82 |
| pIL6_6 | *orf6* | 8210...8842 | 211 | hypothetical protein SMU.1803c | 1 | NP_722122 | 205 (45) | *Streptococcus mutans* |
| pIL6_7 | *orf7* | 9143...10396 | 418 | hypothetical protein SPH_0417 |  | YP_001693828 | 246 (44) | *Streptococcus pneumoniae* |
| pIL6_8 | *cspD* | 11651...11379 | 91 | cold-shock protein CspD |  | YP_796514 | 66 (100) | *L. lactis* subsp. *cremoris* SK11 |
| pIL6_9 | *cspC* | 12064...11858 | 69 | Cold-shock protein CspC |  | YP_796515 | 66 (98) | *L. lactis* subsp. *cremoris* SK11 |
| pIL6_10 | *orf10* | 12808...13095 | 95 | hypothetical protein |  | AAF75621 | 95 (98) | *L. lactis* subsp. *lactis* bv. diacetylactis |
| pIL6_11 | *orf11* | 13198...13575 | 125 | hypothetical protein | 3 | YP_001966474 | 146 (44) | *L. lactis,* pNP40 |
| pIL6_12 | *orf12* | 13579...14061 | 161 | hypothetical protein |  | NP_862031 | 168 (55) | *L. lactis,* pBM02 |
| pIL6_13 | *traG* | 14064...15890 | 577 | conjugation protein TraG | 2 | YP_001966476 | 611 (68) | *L. lactis,* pNP40 |
| pIL6_14 | *orf14* | 15897...16169 | 91 | hypothetical protein | 2 | YP_001966477 | 91 (86) | *L. lactis,* pNP40 |
| pIL6_15 | *orf15* | 16196...16630 | 145 | hypothetical protein | 4 | YP_001966478 | 137 (62) | *L. lactis,* pNP40 |
| pIL6_16 | *orf16* | 16659...17396 | 246 | hypothetical protein | 6 | YP_001966479 | 246 (65) | *L. lactis,* pNP40 |
| pIL6_17 | *orf17* | 17401...17769 | 122 | hypothetical protein | 2 | YP_001966480 | 122 (59) | *L. lactis]* pNP40 |
| pIL6_18 | *traE* | 17705...20083 | 793 | conjugation protein TraE |  | YP_001966481 | 795 (78) | *L. lactis*, pNP40 |
| pIL6_19 | *orf19* | 20099...21979 | 627 | traG-related protein | 1 | NP_072029 | 423 (66) | *Enterococcus faecalis* |
| pIL6_20 | *orf20* | 21986...22582 | 199 | hypothetical protein | 1 | YP_001966484 | 196 (43) | *L. lactis*, pNP40 |
| pIL6_21 | *orf21* | 22566...22910 | 115 | hypothetical protein | 1 | YP_001966485 | 105 (67) | *L. lactis*, pNP40 |
| pIL6_22 | *orf22* | 22910...23134 | 75 | hypothetical protein |  | YP_001966486 | 75 (78) | *L. lactis*, pNP40 |
| pIL6_23 | *traF* | 23127...24347 | 407 | conjugation protein TraF | 1 | YP_001966487 | 413 (57) | *L. lactis*, pNP40 |
| pIL6_24 | *orf24* | 24363...25382 | 340 | hypothetical protein, containing antirestriction domain |  | YP_001966488 | 358 (57) | *L. lactis*, pNP40 |
| pIL6_25 | *orf25* | 25390...25668 | 93 | hypothetical protein |  | NP_862607 | 105 (53) | *L. lactis*, pNP40 |
| pIL6_26 | *mobC* | 25863...26228 | 122 | mobilization protein MobC |  | YP_796489 | 122 (100) | *L. lactis* subsp. *cremoris* SK11 |
| pIL6_27 | *mobD* | 26236...27744 | 503 | relaxase MobD |  | YP_001966491 | 505 (59) | *L. lactis*, pNP40 |

**Table S1g: Putative genes identified on pIL7.**

| Tag | Gene | Position | Length (AA) | Best blast match | Transmembrane domains | Gene Bank accession no. | AA overlap (identity) | Organism, plasmid |
| --- | --- | --- | --- | --- | --- | --- | --- | --- |
| pIL7_1 | *repB* | 26…1177 | 383 | RepB |  | CAA80964 | 385 (96) | *L. lactis* |
| pIL7_2 | *orfX* | 1174…1797 | 206 | hypothetical protein pSRQ700_02 |  | NP_116727 | 209 (56) | *L. lactis,* pSRQ700 |
| pIL7_3 | *hsdS* | 1785…2969 | 394 | HsdS subunit |  | AAC38352 | 395 (97) | *L. lactis,* pIL7 |
| pIL7_4 | *orf4* | 3357…5366 | 669 | dynamin |  | ZP_02184584 | 745 (27) | *Carnobacterium* |
| pIL7_5 | *orf5* | 5522…5884 | 120 | IS-LL6 transposase | 5 | ABA47425 | 409 (100) | *L. lactis* subsp*. cremoris* SK11, pSK11P |
| pIL7_6 | *orf6* | 5902...6741 | 279 | unnamed protein product |  | CAA55220 | 299 (99) | *L. lactis* |
| pIL7_7 | *orf7* | 7342...8280 | 312 | 2-dehydropantoate 2-reductase | 6 | NP_814296 | 312 (99) | *Enterococcus faecalis* |
| pIL7_8 | *orf8* | 8306...9343 | 345 | membrane protein, toxin regulator |  | NP_814295 | 345 (99) | *Enterococcus faecalis* |
| pIL7_9 | *orf9* | 10418…9648 | 256 | transposase, mutator type | 23 | ZP_00603849 | 392 (99) | *Enterococcus faecium* |
| pIL7_10 | *orf10* | 10631…11311 | 226 | transposase |  | NP_569202 | 226 (97) | *Listeria innocua* |
| pIL7_11 | *orf11* | 11636…12673 | 345 | hypothetical protein LACR_C04 |  | YP_796472 | 345(99) | *L. lactis* subsp*. cremoris* SK11, pSK11P |
| pIL7_12 | *orf12* | 12919…13212 | 97 | hypothetical protein |  | NP_862607 | 105 (89) | *L. lactis,* pAH82 |
| pIL7_13 | *orf13* | 13423…13791 | 122 | mobilization protein | 7 | ABA47326 | 122 (87) | *L. lactis* subsp*. cremoris* SK11, pSK11A |
| pIL7_14 | *mobD* | 13796…15313 | 505 | MobD |  | YP_001966491 | 505 (88) | *L. lactis,* pNP40 |
| pIL7_15 | *repC* | 15886…15656 | 76 | RepC |  | NP_858116 | 76 (100) | *L. lactis* subsp. *cremoris,* pHP003 |
| pIL7_16 | *orf16* | 16377…15895 | 160 | RepB family protein | 12 | YP_796485 | 174 (75) | *L. lactis* subsp. *cremoris* SK11, pSK11P |
| pIL7_17 | *orf17* | 16561…16370 | 63 | replication initiator protein |  | YP_796484 | 388 (100) | *L. lactis* subsp*. cremoris* SK11, pSK11P |
| pIL7_18 | *orf18* | 16809…17696 | 295 | transposase AB of ISLL6 |  | YP_001174717 | 379 (84) | *L. lactis,* pGdh442 |
| pIL7_19 | *orf19* | 17751...18368 | 205 | putative transcriptional regulator |  | CAE52354 | 228 (36) | *Streptococcus thermophilus* |
| pIL7_20 | *repA* | 18892...20244 | 450 | replication protein |  | YP_001174679 | 450 (99) | *L. lactis,* pGdh442 |
| pIL7_21 | *orf21* | 20589...21479 | 296 | transposase for insertion sequence element *IS*982B | 2 | YP_001031999 | 296 (89) | *L.* *lactis* subsp. *cremoris* MG1363 |
| pIL7_22 | *parA* | 22007...22765 | 252 | partition protein A |  | YP_001174680 | 252 (99) | *L. lactis,* pGdh442 |
| pIL7_23 | *parB* | 22769...23497 | 242 | partition protein B |  | YP_001174681 | 242 (99) | *L. lactis,* pGdh442 |
| pIL7_24 | *orf24* | 23877…23527 | 116 | hypothetical protein L179764 |  | NP_266743 | 126 (77) | *L. lactis* IL1403 |
| pIL7_25 | *umuC* | 25333…23867 | 488 | UmuC |  | NP_266744 | 488 (89) | *L. lactis* IL1403 |
| pIL7_26 | *hsdR* | 25972…26304 | 110 | type I restriction enzyme R protein |  | NP_266806 | 995 (99) | *L. lactis* IL1403 |
| pIL7_27 | *orf27* | 26556…27097 | 180 | hypothetical protein pDR11B_06 |  | NP_690628 | 180 (96) | *L. lactis* subsp. *lactis* biovar. *diacetylactis,* pDR1-1B |
| pIL7_28 | *orf28* | 27319…27549 | 76 | hypothetical protein pAG6_05 |  | YP_263294 | 92 (97) | *L. lactis* subsp. *cremoris,* pAG6 |
